# Protective Effects of Huangqi Shengmai Yin on Type 1 Diabetes-Induced Cardiomyopathy by Improving Myocardial Lipid Metabolism

**DOI:** 10.1155/2021/5590623

**Published:** 2021-06-18

**Authors:** Zhanhong Cao, Jianheng Pan, Xin Sui, Chunqiu Fang, Na Li, Xiaowei Huang, Xiaobo Qu, Dong Han

**Affiliations:** Changchun University of Chinese Medicine, Changchun 130117, Jilin, China

## Abstract

Diabetic cardiomyopathy (DCM) is one of the many complications of diabetes. DCM leads to cardiac insufficiency and myocardial remodeling and is the main cause of death in diabetic patients. Abnormal lipid metabolism plays an important role in the occurrence and development of DCM. Huangqi Shengmai Yin (HSY) has previously been shown to alleviate signs of heart disease. Here, we investigated whether HSY could improve cardiomyopathy caused by type 1 diabetes mellitus (T1DM) and improve abnormal lipid metabolism in the diabetic heart. Streptozotocin (STZ) was used to establish the T1DM mouse model, and T1DM mice were subsequently treated with HSY for eight weeks. The changes in the cardiac conduction system, histopathology, blood myocardial injury indices, and lipid content and expression of proteins related to lipid metabolism were evaluated. Our results showed that HSY could improve electrocardiogram; decrease the serum levels of CK-MB, LDH, and BNP; alleviate histopathological changes in cardiac tissue; and decrease myocardial lipid content in T1DM mice. These results indicate that HSY has a protective effect against T1DM-induced myocardial injury in mice and that this effect may be related to the improvement in myocardial lipid metabolism.

## 1. Introduction

Diabetes mellitus (DM) is a metabolic disease characterized by chronic hyperglycemia resulting from impaired insulin secretion and/or insulin resistance [1]. By 2030, the global prevalence of DM is expected to rise to 366 million people [2]. The leading cause of death in diabetic patients is DCM, which causes left ventricular dysfunction, myocardial hypertrophy, myocardial fibrosis, and microvascular injury [3, 4]. The etiology of DM is influenced by complex interactions involving both genetic and environmental factors. However, while several cellular processes play a role in the pathogenesis of DCM, including an imbalance in energy metabolism, oxidative stress, mitochondrial dysfunction, endoplasmic reticulum stress, impaired insulin signaling, myocardial fibrosis, and inflammation [5–8], a specific causative factor has not been identified.

An abnormal lipid metabolism in cardiomyocytes is known to play a key role in the occurrence and development of pathological structural and functional changes associated with DCM [9]. In particular, substrate metabolism in cardiomyocytes is altered in DCM; thus, glucose utilization is decreased, and fatty acids (FAs) uptake and utilization are increased [10, 11]. Excess utilization of FAs may cause impaired mitochondrial fatty acids oxidation (FAO), which further increases FAs accumulation [12, 13]. This accumulation of FAs increases myocardial oxygen consumption and mitochondrial dysfunction, leading to cardiomyocyte death and ventricular dysfunction [14]. Therefore, addressing the abnormal metabolism of myocardial substrates by correcting the imbalance between the uptake and oxidation of FAs may be a potential method for the treatment of diabetic cardiomyopathy.

Huangqi Shengmai Yin (HSY) is a Chinese herbal medicine composed of *Astragalus membranaceus*, *Codonopsis pilosula*, *Ophiopogon japonicus*, *Schisandra chinensis*, and *Schisandra sphenanthera*. This modern herbal formula evolved from the basic prescription of Shengmai powder. *Astragalus membranaceus* and *Codonopsis pilosula* belong to energy promotion Chinese medicine, which may promote lipid oxidation and reduce glucagon consumption. Yin-promoting Chinese medicines, *Ophiopogon japonicus* and *Schisandra chinensis*, can facilitate myocardial metabolism, reduce oxygen consumption, and increase ATP and glucagon content [15]. According to previous studies, the main constituents of HSY are Astragaloside, Lobetyolin, Ophiopogonin D, Schisandrin B, and Schisantherin A [16, 17]. HSY has been reported to significantly improve myocardial fibrosis induced by radiation [18]. Astragaloside IV, the main component of *Astragalus membranaceus*, has been demonstrated to relieve myocardial lipid metabolism dysfunction and thus improve myocardial damage induced by type 2 diabetes mellitus [19]. However, whether HSY has a protective effect on T1DM has not been reported. On the basis of the effective regulation of lipid metabolism by each component in the HSY prescription, we speculated that HSY may play a protective role in T1DM by alleviating the dysfunctions in lipid metabolism. In the present study, a mouse model of streptozotocin- (STZ-) induced T1DM was established to investigate the potential protective effect of HSY on myocardial damage caused by diabetes, and its potential mechanism of action was determined.

## 2. Materials and Methods

### 2.1. Drugs and Reagents

HSY (batch no. 190916, approval no. Z36020369) was purchased from Nan Chang Ji Sheng Pharmaceutical Factory (Nanjing, China). STZ was purchased from Sigma-Aldrich (St. Louis, MO, USA). Lactate dehydrogenase (LDH), triglyceride (TG), total cholesterol (TC), high-density lipoprotein cholesterol (HDL), and low-density lipoprotein cholesterol (LDL) assay kits were purchased from Nanjing Jiancheng Bioengineering Institute (Jiangsu, China). Brain natriuretic peptide (BNP) and creatine kinase isoenzyme-MB (CK-MB) were obtained from Jiangsu Enzyme Free Industrial Co., Ltd. Free fatty acid (FFA) ELISA kit was purchased from R & D Systems (Minneapolis, MN, USA). ATP was purchased from the Beyotime Institute of Biotechnology (Shanghai, China). Primary antibodies against PPAR*α*, FGF21, LKB1, CPT1*α*, and Glut4 were purchased from Biosynthesis Biotechnology Co., Ltd. (Beijing, China). Primary antibodies against AMPK*α*, Sirt1, PGC-1*α*, CD36, and *β*-actin were obtained from Proteintech Group, Inc. (Wuhan, China). Primary antibody against p-AMPK*α* was purchased from Affinity Biosciences (OH, USA).

### 2.2. Animals and Treatment

Male ICR mice weighing 20–23 g, were purchased from Changchun Yisi Laboratory Animal Technology Co., Ltd. (Jilin, China). The mice were routinely housed and fed in a standard environment (22 ± 1°C; 12 h light/dark cycles) for one week before experimentation. DM was induced using a single intraperitoneal injection of 160 mg/kg STZ dissolved in 0.1 mol/L citrate buffer (pH 4.5). The same volume of citrate buffer was injected intraperitoneally to Control mice. Mice with fasting blood glucose concentrations ≥16.7 mmol/L and symptoms of polydipsia, polyuria, and polyphagia at two weeks after STZ injection were considered to be diabetic. After establishing the diabetic model, the mice were randomly divided into the following four groups: diabetic mice receiving distilled water (DCM; *n* = 10), diabetic mice treated with HSY at 2 mL/kg/d (DCM + HSY-L; *n* = 10), diabetic mice treated with HSY at 4 mL/kg/d (DCM + HSY-M; *n* = 10), and diabetic mice treated with HSY at 8 mL/kg/d (DCM + HSY-H; *n* = 10). Half of the Control mice received 4 mL/kg/d HSY (Control + HSY-M; *n* = 10), and the other half of Control mice received the same volume of distilled water by intragastric administration daily (Control; *n* = 10). Through acute toxicity experimental studies, we confirmed that the various doses of HSY we used in this experiment are safe (Supplementary 1). After eight weeks, the mice were anesthetized using intraperitoneal injection with pentobarbital (45 mg/kg), and the heart, blood, pancreas, and kidney were harvested for subsequent analysis. All experiments were approved by the Experimental Animal Ethics Committee of Changchun University of Chinese Medicine (approval no. 20190115) and were performed according to the guidelines of the National Institute of Health Guide for the Care and Use of Laboratory Animals.

### 2.3. Blood Sampling and Biochemical Analysis

Blood samples were collected each week from the tail vein of mice fasted overnight, and the blood glucose concentration of the mice was measured using a digital blood glucose meter (Sinocare, Changsha, China). At the end of the experiment, blood was collected from the abdominal aortic vein of mice fasted overnight and then centrifuged at 1500 x g (4°C) for 15 min. The serum was collected and stored at −20°C until analysis. The serum levels of CK-MB, LDH, BNP, TG, TC, HDL, LDL, and FFA were measured according to the kit instructions.

### 2.4. Electrocardiography Measurements

The mice were anesthetized using intraperitoneal injection with pentobarbital (45 mg/kg) and then fixed on a table in the supine position. Subcutaneous needle electrodes were connected to the mice for the limb lead at position II and electrocardiograms were recorded using the Biopac MP150 data acquisition systems (Biopac Systems, Inc., California, USA).

### 2.5. Determination of Heart Weight Index

At the end of the experiment, the body weight and heart weight of each mouse were measured and recorded. The hearts were harvested, rinsed in phosphate-buffered saline, drained with filter paper, and then weighed. The degree of cardiac hypertrophy was evaluated by calculating the heart weight index [HWI = HW/body weight (BW)].

### 2.6. Histopathological Examinations

After fixation with 4% paraformaldehyde for 24 h, mice hearts were embedded in paraffin and sectioned at 5 *μ*m. Sections were then dewaxed, hydrated, and stained with hematoxylin-eosin (HE). The sections were then observed using an optical microscope and images were obtained. Cardiac fibrosis was evaluated by Masson staining. The sections were incubated with Masson's trichrome stain following routine methods, and the degree of myocardial fibrosis was quantitatively analyzed using the Image-Pro Plus 6.0 image analysis software (Media Cybernetics, Rockville, MD, USA).

Cardiac lipid accumulation was evaluated by Oil Red O staining. Cryosections (10 *μ*m) from OCT-embedded heart tissues were washed with 70% ethanol for 5 s. The slides were then immersed in an Oil Red O working solution for 8 min. After washing with 70% ethanol to remove excess dye, the slides were counterstained with hematoxylin for 2 min. The degree of lipid accumulation in myocardial tissue was quantitatively analyzed using Image-Pro Plus 6.0 software.

### 2.7. Transmission Electron Microscopy (TEM)

The myocardial tissue was fixed in 4% glutaraldehyde, dehydrated using a gradient ethanol series, and embedded in Eponate 12 epoxy resin. After sectioning, the samples were double-stained with uranyl acetate and lead citrate. Lastly, ultrastructural changes in cardiomyocytes were observed by TEM.

### 2.8. Immunohistochemistry

The 5 *μ*m paraffin sections of the myocardial tissue were dewaxed, hydrated, and incubated with endogenous peroxidase blockers for 10 min. The antigens were retrieved by microwave heating. After blocking with BSA for 30 min, the sections were incubated with PPAR*α*, COL I, and COL III (1:100) antibodies overnight at 4°C. Goat anti-rabbit IgG was then added dropwise, and the sections were incubated for 30 min. After incubation with SABC for 30 min, DAB chromogenic solution was added to evaluate the degree of staining. The sections were then counterstained with hematoxylin for 2 min. The histochemistry score (H-Score) was subsequently calculated using Image-Pro Plus software 6.0 and used as an indicator of the level of protein. H-Score = ratio of strong-positive ^*∗*^ 3 + ratio of moderate-positive ^*∗*^ 2 + ratio of weak-positive ^*∗*^ 3.

### 2.9. Determination of the ATP Content in the Myocardial Tissue

The ATP content in the myocardial tissue was measured according to the manufacturer's instructions.

### 2.10. Western Blotting

The left ventricular myocardial tissue was lysed in ice-cold RIPA buffer (Beijing Ding Guo Chang Sheng Biotechnology Co., Ltd.) and protein was extracted. The concentration of protein was quantified using the BCA Protein Assay kit. Equal amounts of protein were then separated using a 10% SDS-PAGE gel. The resolved proteins were then transferred from the gel to a polyvinylidene difluoride membrane (Millipore Co., Ltd., USA). The membrane was blocked with 5% nonfat milk at 37°C for 2 h, and then incubated with primary antibody overnight at 4°C. The primary antibodies used were as follows: anti-PPAR*α* (1:500), anti-FGF21 (1:500), anti-LKB1 (1:500), anti-p-AMPK*α* (Thr172) (1:500), anti-GLUT4 (1:500), anti-CPT1*α* (1:500), anti-AMPK*α* (1:1000), anti-Sirt1 (1:1000), anti-CD36 (1:1000), anti-PGC1*α* (1:1000), and anti-*β*-actin (1:1000). The membranes were then washed with TBST and incubated with a horseradish peroxidase-conjugated secondary antibody (1:10000) at 37°C for 1 h. Finally, antibody binding was visualized using a chemiluminescent imaging system and band density was quantitatively analyzed using Image-J software (NIH, Bethesda, MD, USA).

### 2.11. Statistical Analyses

All experimental results were analyzed using GraphPad Prism 8 (GraphPad Software, San Diego, CA, USA). The results are presented as mean ± standard deviation (SD) of continuous variables. Student's two-tailed *t*-test was used for comparison between two groups to determine statistical significance, and one-way analysis of variance was used for comparison among multiple groups. Results with a *P* value of <0.05 were considered statistically significant.

## 3. Results

### 3.1. Pathological Characteristics of Diabetic Mice

**Figure 1:**
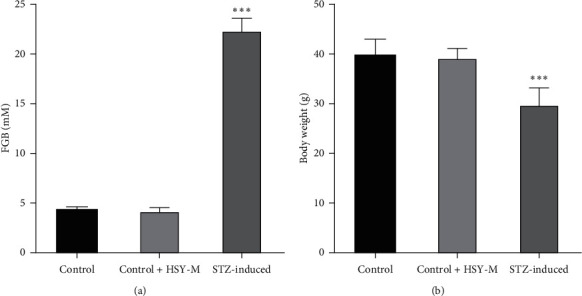
Pathological characteristics of diabetic mice. (a) Fasting blood glucose. (b) Body weight. Data are expressed as mean ± SD. ^*∗∗∗*^*P* < 0.001 versus the Control group.

Two weeks after STZ administration, a significant increase in fasting blood glucose level was observed in comparison with that in the Control and Control + HSY-M groups (Figure 1(a)). In addition, body weight decreased (Figure 1(b)) and symptoms of polydipsia, polyuria, and polyphagia were observed. These results demonstrate that the T1DM model induced by STZ was successfully established.

### 3.2. Effects of HSY on Body Weight and Fasting Blood Glucose

**Figure 2:**
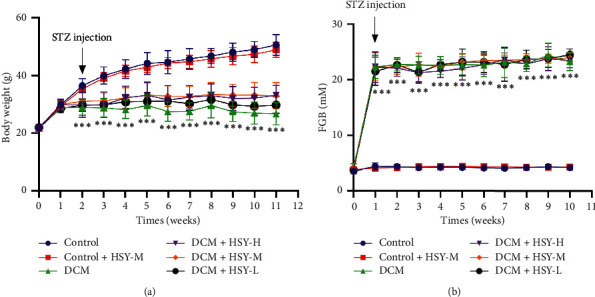
Effects of HSY on body weight and fasting blood glucose in DCM mice. Data are expressed as mean ± SD. ^*∗∗∗*^*P* < 0.001 versus the Control group.

The obvious characteristics of diabetes are a relative decrease in body weight gain and an increase in blood glucose. As shown in Figure 2, the body weight of the Control group mice increased steadily, and blood glucose levels were normal. There was no significant difference between the Control and Control + HSY-M groups. In contrast, the body weight increased comparatively slowly, and blood glucose level significantly increased in the DCM group. Following HSY treatment, body weight gain increased compared with the DCM group, but there was no statistical significance. However, the blood glucose level remained high. Thus, HSY treatment may have no effect on the blood glucose level.

### 3.3. Effect of HSY Treatment on Myocardial Injury and Myocardial Hypertrophy

**Figure 3:**
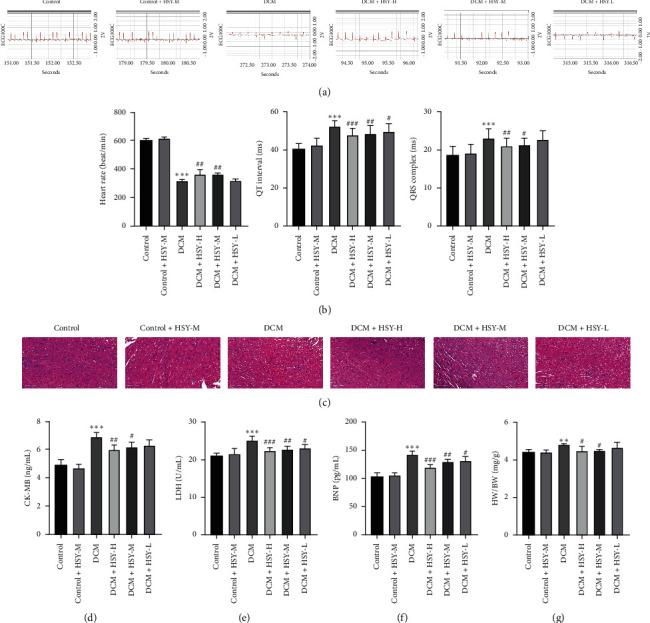
Effect of HSY treatment on myocardial injury and myocardial hypertrophy. (a) Representative images of ECG. (b) ECG parameters. (c) HE staining of heart tissues (scale bar = 100 *μ*m). (d) Serum level of CK-MB. (e) Serum level of LDH. (f) Serum level of BNP. (g) Ratio of heart weight to body weight. Data are expressed as mean ± SD. ^*∗*^*P* < 0.05, ^*∗∗*^*P* < 0.01, and ^*∗∗∗*^*P* < 0.001 versus the Control group; ^#^*P* < 0.05, ^##^*P* < 0.01, and ^###^*P* < 0.01 versus the DCM group.

The effects of HSY on the ECG parameters are shown in Figure 3(a). There was no significant difference between the Control and Control + HSY-M groups. The heart rate decreased and the QT interval and QRS complex were prolonged in the DCM group compared with those in the Control group (Figure 3(b)). After HSY treatment for eight weeks, the heart rate significantly increased and the QT interval and QRS complex were shortened in comparison with those in the DCM group.

Myocardial HE staining was used to evaluate the effect of HSY on the myocardial structure. In the Control and Control + HSY-M groups (Figure 3(c), the morphology of myocardial cells was normal, myocardial fibers were arranged in an orderly fashion, and the cell boundary was clearly delineated. In the DCM group, myocardial fibers were disordered, myocardial cell nuclei were dissolved or absent, and the boundary between cells was blurred. These changes were accompanied by inflammatory cell infiltration. Compared with the DCM group, HSY treatment for eight weeks significantly alleviated these pathological changes.

Cardiac markers in blood are the mainstay for cardiac injury diagnosis. There was no significant difference between the Control and Control + HSY-M groups. A significant increase in the levels of cardiac injury markers was observed in the DCM group compared with the Control group (Figures 3(d) and 3(e)). The serum levels of CK-MB and LDH were significantly lower than those in the DCM group after treatment with HSY.

The degree of cardiac hypertrophy was evaluated by the serum BNP level and heart weight index. As shown in Figures 3(f) and 3(g), there was no significant difference in the BNP level and HW/BW between the Control and Control + HSY-M groups, while the BNP level and HW/BW of the DCM group significantly increased in comparison with the Control group. Compared with the DCM group, HSY treatment significantly decreased the BNP level and HW/BW.

### 3.4. Effect of  HSY Treatment on Lipid Metabolism Dysfunction

**Figure 4:**
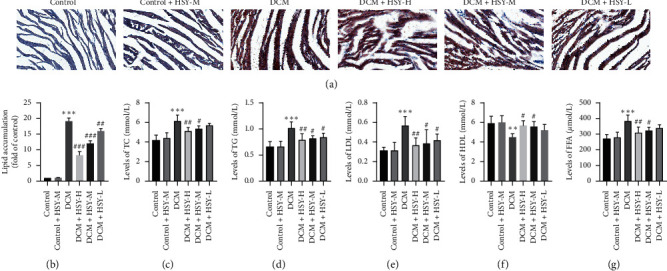
Effect of HSY treatment on lipid metabolism dysfunction in DCM mice. (a) Oil Red O staining of myocardial tissues (scale bar = 50 *μ*m). (b) quantitative analysis of Oil Red O staining. (c) Serum level of TC. (d) Serum level of TG. (e) Serum level of LDL. (f) Serum level of HDL. (g) Serum level of FFA. Data are expressed as mean ± SD. ^*∗*^*P* < 0.05, ^*∗∗*^*P* < 0.01, and ^*∗∗∗*^*P* < 0.001 versus the Control group; ^#^*P* < 0.05, ^##^*P* < 0.01, and ^###^*P* < 0.01 versus the DCM group.

Dysfunction in lipid metabolism plays an important role in the occurrence and development of DCM. To evaluate dysfunction in lipid metabolism, cardiac lipid accumulation was measured by Oil Red O staining. There was no significant difference between the Control and Control + HSY-M groups. Myocardial lipid content significantly increased in the DCM group compared with the Control group (Figures 4(a) and 4(b)). However, HSY treatment for eight weeks significantly reduced the lipid content in the myocardium compared with the DCM group. In addition, the serum lipid metabolism profile was evaluated. Levels of TC, TG, LDL (Figures 4(c)–4(e)), and FFA (Figure 4(g)) significantly increased in the DCM group compared with the Control group, while the level of HDL (Figure 4(f)) significantly decreased. HSY treatment significantly decreased the levels of TC, TG, LDL, and FFA and increased the level of HDL in comparison with the DCM group. There was no significant difference between the Control and Control + HSY-M groups. These results indicate that HSY can alleviate the dysfunction in lipid metabolism induced by DCM.

### 3.5. Effects of HSY Treatment on Structural Damage and Dysfunction of Myocardial Mitochondria

**Figure 5:**
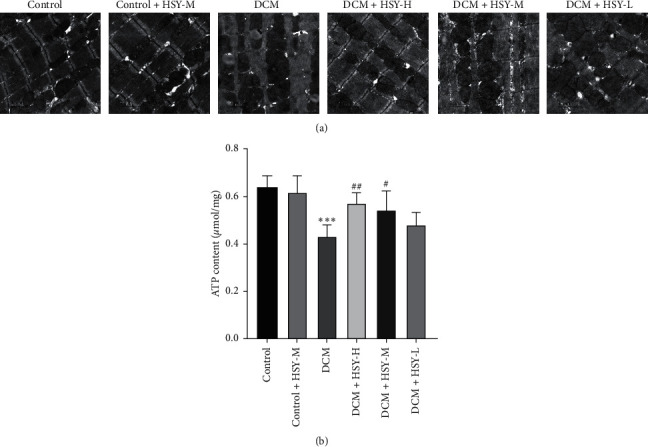
Effect of HSY treatment on structural damage and dysfunction of myocardial mitochondria in DCM mice. (a) TEM images of the mitochondrial structure (scale bar = 1 *μ*m). (b) ATP content of myocardial tissues. Data are expressed as mean ± SD. ^*∗*^*P* < 0.05, ^*∗∗*^*P* < 0.01, and ^*∗∗∗*^*P* < 0.001 versus the Control group; ^#^*P* < 0.05, ^##^*P* < 0.01 versus the DCM group.

Excessive accumulation of FAs can lead to structural damage and dysfunction of mitochondria. The ultrastructural morphology of cardiomyocyte mitochondria and lipid droplet accumulation therein were observed by TEM. As shown in Figure 5(a), there was no significant difference between the Control and Control + HSY-M groups. The DCM group demonstrated a disordered mitochondrial myofilament arrangement, blurred sarcomere, incomplete mitochondrial membrane structure, extensive cristae loss, and excessive lipid droplet distribution in comparison with the Control group. Cardiomyocyte ATP content in the DCM group also significantly decreased compared with the Control group (Figure 5(b)). These results reveal that excessive accumulation of FAs in cardiomyocytes is associated with structural damage and dysfunction of mitochondria. These characteristics were substantially alleviated by HSY treatment. These results indicate that HSY can protect cardiomyocytes from mitochondrial structural damage and dysfunction induced by DCM.

### 3.6. Effect of HSY Treatment on Myocardial Fibrosis

**Figure 6:**
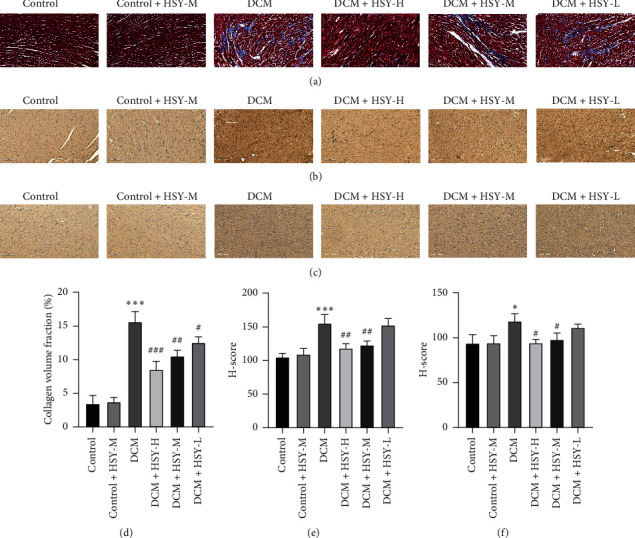
Effect of HSY treatment on myocardial fibrosis in DCM mice. (a) Masson staining of myocardial tissues (scale bar = 100 *μ*m). Representative images of immunohistochemistry for Col I (b) and Col III (c) protein expression in myocardial tissues (scale bar = 100 *μ*m). (d) Quantitative analysis of Masson staining. Quantitative analysis of Col I (e) and Col III (f). Data are expressed as mean ± SD. ^*∗*^*P* < 0.05, ^*∗∗*^*P* < 0.01, and ^*∗∗∗*^*P* < 0.001 versus the Control group; ^#^*P* < 0.05, ^##^*P* < 0.01, and ^###^*P* < 0.001 versus the DCM group.

Myocardial fibrosis was evaluated by Masson staining (Figure 6(a)). There was no significant difference in the degree of fibrosis between the Control and Control + HSY-M groups, while the degree of fibrosis in the DCM group significantly increased compared with the Control group, indicating that myocardial fibrosis may be an important pathological process of DCM. HSY treatment significantly decreased the degree of myocardial fibrosis in comparison with the DCM group. To further explore the effect of HSY on myocardial fibrosis, the expression of two fibrosis factors (COL I and COL III) was measured using immunohistochemical analysis. Expression of COL I and COL III was not significantly different between the Control and Control + HSY-M groups. Compared with the Control group, the expression of COL I and COL III significantly increased in the DCM group (Figures 6(b), and 6(c)). However, HSY treatment significantly decreased the expression of COL I and COL III in comparison with the DCM group. These results indicate that HSY can reverse myocardial fibrosis induced by DCM.

### 3.7. Effect of HSY Treatment on Expression of PPAR*α*

**Figure 7:**
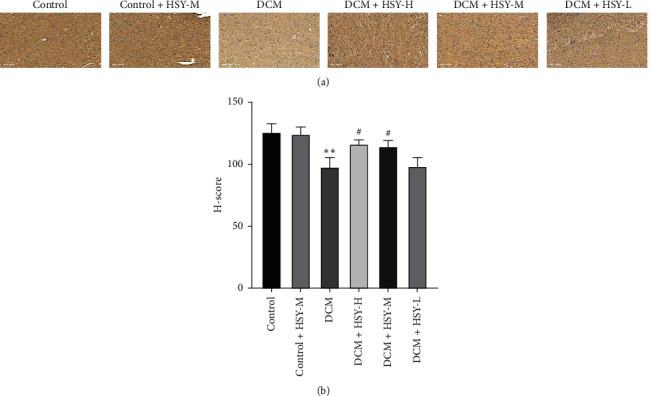
Effect of HSY treatment on expression of PPAR*α* in DCM mice. (a) Representative images of immunohistochemistry for PPAR*α* protein expression in myocardial tissues. (scale bar = 100 *μ*m). (b) Quantitative analysis of PPAR*α* protein expression. Data are expressed as mean ± SD. ^*∗*^*P* < 0.05 and ^*∗∗*^*P* < 0.01 versus the Control group; ^#^*P* < 0.05 and ^##^*P* < 0.01 versus the DCM group.

The expression level of PPAR*α* was measured using immunohistochemical analysis. As shown in Figure 7, there was no significant difference in the expression level of PPAR*α* between the Control and Control + HSY-M groups. PPAR*α* expression in the DCM group significantly decreased in comparison with the Control group. HSY treatment increased PPAR*α* expression in comparison with the DCM group. This result indicates that HSY can increase PPAR*α* expression in DCM, and this result was also confirmed by western blotting.

### 3.8. Effect of HSY Treatment on the Expression of Proteins That Affect Lipid Metabolism

**Figure 8:**
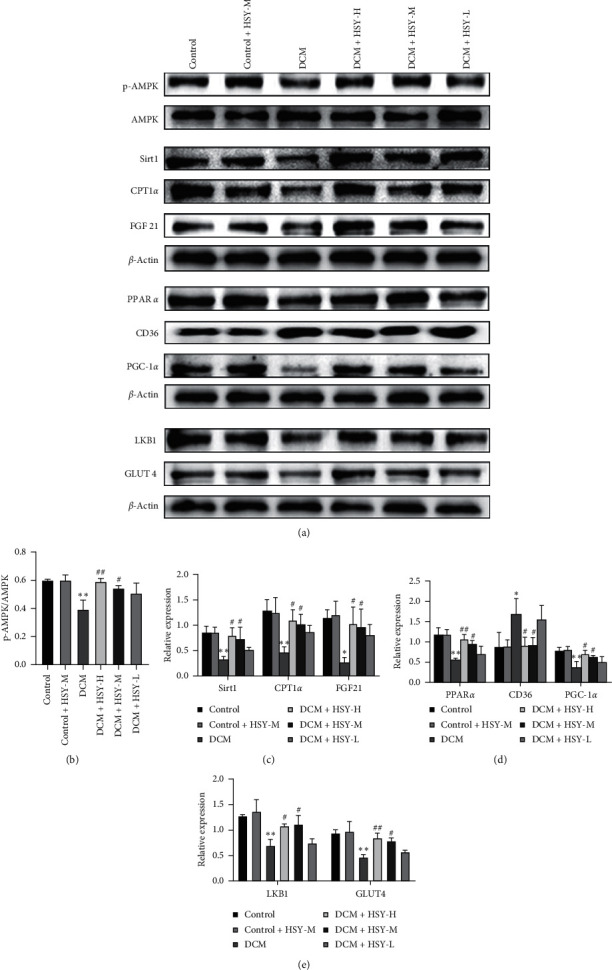
Effect of HSY treatment on the expression of proteins that affect lipid metabolism in DCM mice. (a) Expression levels of related proteins in myocardial tissue. ((b)–(e)) Expression levels of the related proteins were calculated. *β*-Actin was used as an internal control. Data are expressed as mean ± SD. ^*∗*^*P* < 0.05, ^*∗∗*^*P* < 0.01, and ^*∗∗∗*^*P* < 0.01 versus the Control group; ^#^*P* < 0.05, ^##^*P* < 0.01, and ^###^*P* < 0.01 versus the DCM group.

To explore the possible mechanisms through which HSY regulates lipid metabolism, we evaluated the expression of key proteins involved in lipid uptake, synthesis, and oxidation. As shown in Figure 8, there was no statistical significance in the expression of proteins between the Control and Control + HSY-M groups. Expression of PPAR*α*, FGF21, p-AMPK*α*, LKB1, Sirt1, PGC-1*α*, CPT1*α*, and Glut4 in the DCM group significantly decreased, while the expression of CD36 significantly increased in comparison with the Control group. These results suggest that DCM leads to decreased glucose uptake and oxidation, increased lipid uptake, and impaired FAO. HSY treatment significantly increased the expression of PPAR*α*, FGF21, p-AMPK*α*, LKB1, Sirt1, PGC-1*α*, CPT1*α*, and Glut4 and decreased the expression of CD36 compared with the DCM group. These results indicate that HSY can improve the abnormal expression of proteins associated with lipid metabolic pathways, thereby reducing myocardial lipid accumulation induced by DCM.

## 4. Discussion

Diabetic cardiomyopathy is the main cause of morbidity and death in diabetic patients [20, 21]. However, effective drugs for the treatment of this condition are limited. In this study, we used STZ to successfully establish a T1DM mouse model. Our results show that HSY can improve DCM by ameliorating the lipid metabolism disorder of this model mouse.

Diabetic patients have a high incidence of diabetic cardiomyopathy, which is characterized by complex changes in the mechanical and electrical properties of the heart [22]. In this study, there was a decrease in the heart rate and prolongation of the QT interval and QRS complex in T1DM mice, indicating the dysfunction of the cardiac conduction system. These findings are consistent with those of the results of previous research [23]. However, HSY significantly attenuated the ECG abnormalities.

In STZ-induced DCM animal models, elevated blood glucose and lipid levels were associated with increased myocardial injury indices in the blood [24]. CK-MB and LDH serve as cardiac biomarkers to detect cardiac injury. In this study, the levels of CK-MB and LDH in the DCM group were significantly increased. Furthermore, HE staining revealed that the myocardial fibers were disordered, the myocardial cell nuclei were dissolved or lost, and the boundary between the cells was blurred in the DCM group. These changes were also accompanied by inflammatory cell infiltration. The results of histopathology and cardiac marker enzymes confirmed that HSY alleviated cardiac injury caused by DCM.

Myocardial hypertrophy and fibrosis are among the most representative changes in DCM [25, 26]. In DCM, the dynamic balance between synthesis and deposition of the myocardial extracellular matrix is disturbed, and excessive deposition of collagen and an imbalance in the collagen ratio lead to cardiac remodeling [27–29]. BNP is among the most relevant molecular markers of cardiac hypertrophy [30], and the level of BNP increased in the DCM group. The observed increase in the HW/BW ratio in the DCM group may be related to both body weight loss and an increase in the myocardial extracellular matrix. Moreover, Masson staining revealed obvious myocardial interstitial collagen deposition and a comparatively high degree of fibrosis in the DCM group. COL I and COL III, the two main components of ECM, play a major role in maintaining the structure and function of the heart [31]. Diabetes-related myocardial fibrosis is associated with the accumulation of COL I and COL III [32]. Our immunohistochemistry results confirmed that the expression of collagen I/III significantly increased in the DCM group. The results of this study indicated that HSY improves myocardial hypertrophy and fibrosis induced by DCM.

According to previous studies, cardiac insulin signaling in T1DM patients is diminished, and so the heart relies more on FAO to obtain energy; thus, myocardial metabolism is transformed from glucose oxidation to FAO [33–35]. However, lipid uptake ultimately exceeds lipid clearance, resulting in significant lipid accumulation in the diabetic heart. Oil Red O staining results demonstrate that myocardial lipid accumulation was significantly increased in T1DM mice. The serum levels of TC, TG, LDL, and FFA also increased and the level of HDL decreased. HSY treatment significantly reduced the level of myocardial lipid accumulation. In addition, TEM confirmed that HSY could reduce lipid accumulation and associated injuries in myocardial mitochondria. To further evaluate the status of mitochondria, we measured the content of ATP in the myocardium and found that HSY could reverse a decrease in ATP content induced by T1DM. Thus, HSY may protect myocardial mitochondria by reducing myocardial lipid accumulation. The above results confirm our hypothesis that HSY can alleviate the effects of abnormal lipid metabolism induced by T1DM.

Next, we focused on the protective action mechanism of HSY in T1DM. Mitochondrial FAO is the main pathway involved in FAs catabolism. Several studies have confirmed that a further increase in FAO can reduce lipid accumulation and lipid toxicity [36–38]. PPAR*α*, the main regulator of glycolipid homeostasis, can regulate lipid metabolism by improving lipid absorption and oxidation [39, 40]. Decreased PPAR*α* expression and continuous exposure to elevated levels of FFA increase lipid accumulation in DCM [41]. PPAR*α* activation can upregulate mitochondrial FAO and promote FAs catabolism [42]. The expression levels of several additional proteins, including FGF21, p-AMPK*α*, LKB1, Sirt1, PGC-1*α*, CPT1*α*, and Glut4, also affect lipid metabolism and energy homeostasis.

FGF21 is a downstream mediator of PPAR*α* and plays an important role in energy homeostasis [42]. FGF21 deficiency increases lipid uptake and decreases FAO in diabetic mice, resulting in lipid accumulation in the heart [43, 44]. AMPK plays an important role in regulating the energy balance of cells. Activation of AMPK increases glucose uptake and FAO in metabolic diseases such as diabetes [45, 46]. LKB1 is the main regulator of AMPK activation, and it can directly phosphorylate AMPK Thr-172 and activate its enzyme activity [47]. In addition, specific deletion of cardiac LKB1 can lead to myocardial fibrosis and cardiac dysfunction [48].

Sirt1 is considered a potential target for the treatment of DCM, and its decreased expression leads to impaired insulin signaling and abnormal mitochondrial dynamics [49]. Sirt1 directly binds PPAR*α* and it can promote the interaction between PGC-1*α* and PPAR*α* and regulate cardiac metabolism [50]. PGC-1*α* can upregulate the expression of various genes involved in FAO and the tricarboxylic acid cycle and coactivate PPAR*α* to enhance FAO, which is positively regulated by AMPK [51, 52]. Therefore, activation of PGC-1*α* can prevent the occurrence of diabetes [53]. The expression level of CPT1*α*, a key enzyme that regulates the oxidation of FAs entry into mitochondria, also decreased in the diabetic heart [54]. The drop may cause impaired mitochondrial FAO.

Our results provide evidence that FAO is impaired in the heart of T1DM mice, resulting in lipid accumulation. Moreover, we show that HSY treatment can significantly reduce myocardial lipid accumulation. A potential mechanism of HSY action may improve myocardial FAO, reduce myocardial lipid accumulation, and increase glucose utilization through AMPK/PPAR*α*/FGF21 signal pathway and ultimately play a protective role in myocardial injury induced by T1DM.

CD36 mediates the transportation and utilization of FAs in the heart [55]. Upon the development of diabetes, cardiomyocytes absorb more FFA to provide energy through FAs transferase CD36; however, excessive FAs uptake damages mitochondrial FAO, leading to mitochondrial dysfunction and lipid accumulation [56]. According to our study, HSY both increased lipid clearance and decreased CD36 expression in the heart of T1DM mice. Glucose uptake is mediated by the glucose transporter, GLUT4. Insulin resistance and accumulation of excessive FAs may negatively affect the expression and translocation of GLUT4 in cardiomyocytes and thus further reduce glucose uptake and oxidation in cardiomyocytes. GLUT4 is positively regulated by AMPK [57, 58]. According to our study, HSY can increase the expression of GLUT4 in the myocardium of T1DM mice. Therefore, HSY treatment also can reduce myocardial lipid uptake and increase myocardial glucose uptake and utilization.

Taken together, our results show that the HSY has a beneficial effect on improving myocardial injury induced by T1DM, and its mechanism may be closely related to improving myocardial lipid metabolism. In future studies, we aim to further investigate the protective effect and mechanism of action of HSY in T1DM through in *vitro* experiments.

## Data Availability

The data used to support the findings of this study are available from the corresponding author upon request.

## Supplementary Materials

Supplementary Materials Supplementary 1. Acute Toxicity Testing of Huangqi Shengmai Yin. Mice were intragastrically administered Huangqi Shengmai Yin 3 times at a dose of 40 mL/kg, and the cumulative dose was 120 mL/kg, which is equivalent to 280 times daily clinical dose (30 mL/70 kg/day). Through observation for 14 days, no adverse reactions and death.
