# Supplementary material for: Protective Effects of Huangqi Shengmai Yin on Type 1 Diabetes-Induced Cardiomyopathy by Improving Myocardial Lipid Metabolism
Source: Evid Based Complement Alternat Med. 2021 Jun 18;2021:5590623. doi: 10.1155/2021/5590623 (PMC8238573; doi:10.1155/2021/5590623)
Supplement: Supplementary Materials — Supplementary 1. Acute Toxicity Testing of Huangqi Shengmai Yin. Mice were intragastrically administered Huangqi Shengmai Yin 3 times at a dose of 40 mL/kg, and the cumulative dose was 120 mL/kg, which is equivalent to 280 times daily clinical dose (30 mL/70 kg/day). Through observation for 14 days, no adverse reactions and death. [file 5590623.f1.doc]

Acute Toxicity Testing of Huangqi Shengmai Yin

**Abstract:** According to “Technical Guidelines for Acute Toxicity Research of Traditional Chinese Medicine and Natural Medicine”, the acute toxicity of Huangqi Shengmai Yin was observed by intragastric administration in mice. Since the LD_50_ could not be measured, maximum dose was determined. Mice were given Huangqi Shengmai Yin with a maximum volume of 40ml/kg each time, 3 times a day, and the cumulative dose was 120 mL/kg, which is equivalent to 280 times daily clinical dose (30 mL/70 kg/day). No adverse reactions and deaths were found.

**1 Experimental drug**

Huangqi Shengmai Yin（HSY） was purchased from Nan Chang Ji Sheng Pharmaceutical Factory (Nanjing, China). specification: 10 mL/bottle, batch no. 190916, approval number: Z36020369.

**2 Animals**

Male ICR mice weighing 18-22 g, were purchased from Changchun Yisi Laboratory Animal Technology Co., Ltd. (Jilin, China). Mice were routinely housed and fed in a standard environment (22±1°C; 12 h light/ dark cycles) for 3 days prior to experimentation.

**3 methods**

Ten mice were given once with the maximum volume (40 mL/kg). After 7 days of observation, no adverse reactions and deaths were found. Because the LD_50_ could not be calculated, we carried out the maximum gavage dose measurement.

After fasting for 12h, 25 mice were given 3 times by gavage with a maximum volume of 40 mL/kg, and the cumulative dose was 120 mL/kg, which is equivalent to 280 times daily clinical dose (30 mL/70 kg/day). The other 10 mice were given an equal volume of distilled water as control group. The animal's coat color, voluntary activities, breathing, diet, weight and death were observed daily for 14 consecutive days.

**4 results**

After the administration, 25 mice had no obvious adverse reactions and died. There is no difference in weight between the blank control group and the Huangqi Shengmai Yin group (Fig. 1). The mental state was good, the coat was white and shiny, and the stool and urine were normal. It is found that the maximum gavage dose of Huangqi Shengmai Yin to mice is higher than 120 mL/kg, which is equivalent to 280 times daily clinical dose (30 mL/70 kg/day).

**5 Conclusion**

Mice were intragastrically administered Huangqi Shengmai Yin 3 times at a dose of 40 mL/kg, and the cumulative dose was 120 mL/kg, which is equivalent to 280 times daily clinical dose (30 mL/70 kg/day). Through observation for 14 days, no adverse reactions and death.


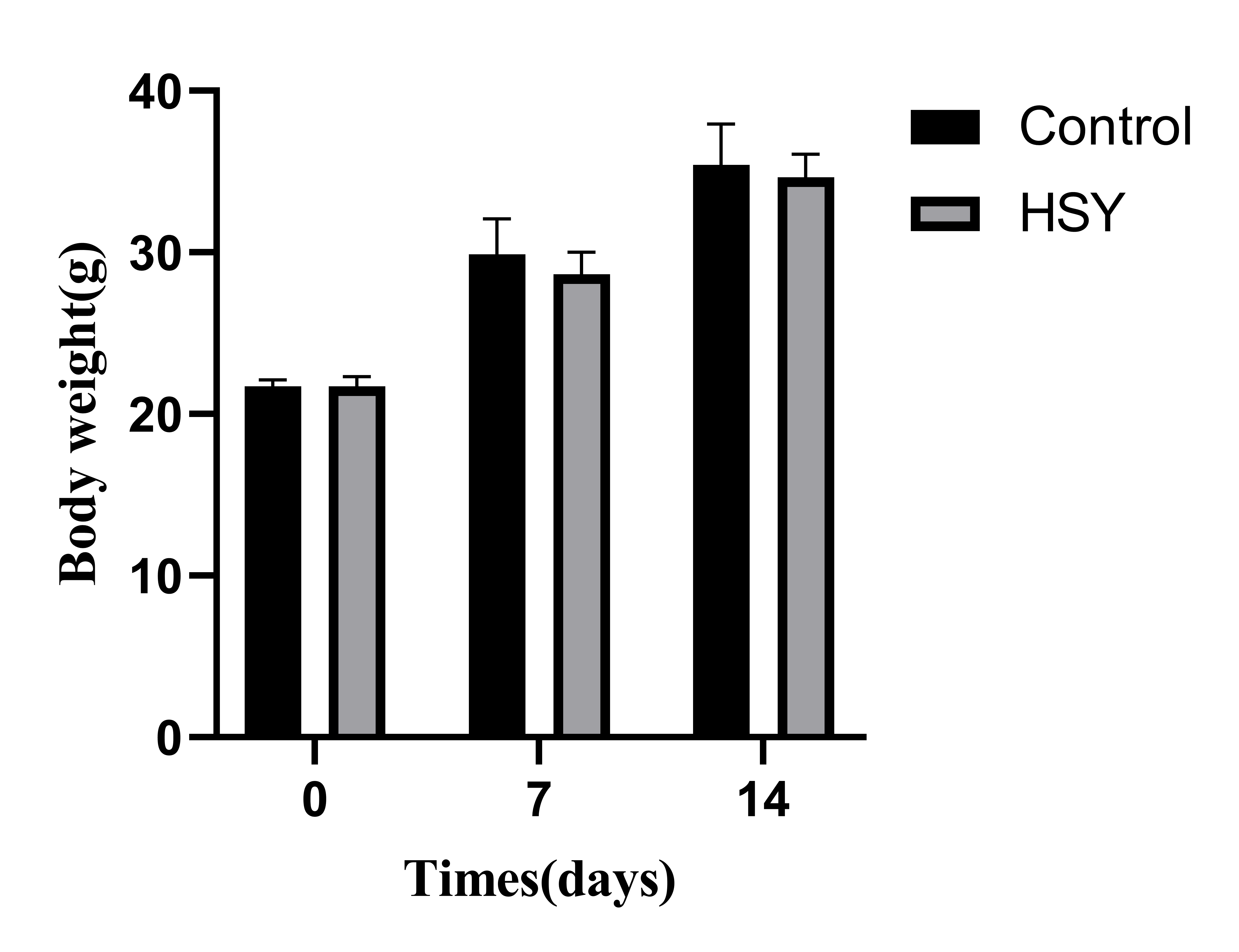


Figure 1
